# Crosstalk between IL-6/JAK/STAT3 and TNF-α/NF-κB signaling in colitis-associated colorectal cancer: epigenetic reprogramming, evidence boundaries, and opportunities for disease management

**DOI:** 10.3389/fimmu.2026.1979583

**Published:** 2026-10-26

**Authors:** Jin Yuan, Jinping Liao, Fen Li, Denglu Xie, Zhiheng Zuo, Di Zhang, Tianwu Zhang

**Affiliations:** Pu’er Traditional Chinese Medical Hospital, Pu’er, Yunnan, China

**Keywords:** biomarker validation, colitis-associated colorectal cancer, Crohn’s colitis, DNA methylation, epigenetic memory, IL-6, inflammatory bowel disease, JAK/STAT3

## Abstract

Colitis-associated colorectal cancer (CAC) arises through the interaction of chronic mucosal inflammation, genetic evolution and durable changes in gene regulation. Among the inflammatory networks implicated in experimental and human studies, interleukin-6 (IL-6)/Janus kinase (JAK)/signal transducer and activator of transcription 3 (STAT3) and tumor necrosis factor-alpha (TNF-α)/nuclear factor-kappaB (NF-κB) provide mechanistically informative routes by which cytokine exposure can influence DNA methylation, chromatin regulation and non-coding RNA programs. The best-supported examples include IL-6-associated DNMT1 activity and SOCS3 promoter hypermethylation, NF-κB p65/DNMT3A-associated miR-148a methylation, and STAT3-dependent miR-214 induction. These circuits should be regarded as representative mechanisms supported in specific model systems rather than universal initiating pathways in all human CAC lesions. Recent work further demonstrates that colonic stem cells can retain chromatin accessibility changes after experimental colitis resolves, providing direct evidence for durable inflammatory epigenetic memory. This review therefore separates evidence from human CAC or dysplasia, non-neoplastic inflammatory bowel disease (IBD) mucosa, inflammation-driven animal models, sporadic colorectal cancer (CRC) and cell-line systems. We integrate cytokine-linked epigenetic mechanisms with genetic and clonal evolution, define field cancerization operationally, and distinguish persistent epigenetic memory from transient inflammatory transcription. The translational sections are framed as an outlook: no epigenetic biomarker or epigenetic drug is currently established for CAC surveillance or prevention. We summarize the validation requirements for tissue, blood and stool biomarkers; classify therapeutic concepts by evidence level; and propose a patient-level roadmap for single-cell and spatial studies. The resulting framework emphasizes evidence-graded mechanisms and prospective validation rather than assuming that biologically plausible signals are clinically actionable.

## 1 Introduction

Chronic intestinal inflammation is a well-established risk context for colorectal neoplasia. In ulcerative colitis (UC) and Crohn’s disease involving the colon, risk is shaped by cumulative inflammatory burden, disease duration and extent, primary sclerosing cholangitis, prior dysplasia and other clinical modifiers (1–4). In this review, CAC denotes colorectal dysplasia or carcinoma arising in a chronically inflamed IBD colon; when evidence is restricted to UC-associated neoplasia, we state this explicitly. Sporadic CRC is discussed only as indirect supporting evidence and is not treated as equivalent to CAC.

CAC cannot be reduced to either a mutation-first or an epigenetic-only model. TP53 alterations may occur early, APC alterations can occur later or at lower frequency than in the conventional adenoma-carcinoma sequence, and copy-number change, inflammation-associated mutagenesis and clonal selection contribute to progression (5, 6). These genetic events occur within an inflammatory ecosystem characterized by repeated epithelial injury, regeneration, cytokine exposure and immune-cell recruitment. Epigenetic plasticity can influence which clones survive these cycles, whereas genetic change can stabilize or reshape the same cell states. We therefore use a genetic-epigenetic co-evolution framework rather than treating genetic lesions as secondary background events.

IL-6/JAK/STAT3 and TNF-α/NF-κB are emphasized because their roles in inflammation-associated tumorigenesis are experimentally tractable and because both pathways interface with chromatin-modifying machinery. IL-6/gp130/STAT3 signaling supports epithelial survival and tumor development in colitis-associated models (7, 8), whereas epithelial IKK/NF-κB and TNF signaling contribute to tumor-promoting inflammation in mouse CAC models (9, 10). These findings do not establish either pathway as a universal initiating driver of human CAC. Instead, they provide specific examples of how repeated inflammatory signaling may influence durable regulatory states.

The review is organized in two parts. Sections 3–7 synthesize published mechanistic evidence and explicitly label the relevant model system. Section 8 is a translational outlook covering biomarker development, therapeutic opportunities and new technologies. This separation is intended to distinguish what has been demonstrated from what remains inference or future hypothesis.

## 2 Review strategy, scope and evidence framework

### 2.1 Literature search and study selection

This narrative review used a structured PubMed/MEDLINE search updated through 22 September 2026. Search concepts combined terms for disease (“colitis-associated colorectal cancer”, “ulcerative colitis-associated colorectal cancer”, “IBD-associated colorectal cancer”, “colitis-associated cancer”) with pathway terms (“IL-6”, “JAK”, “STAT3”, “TNF”, “NF-kappaB”) and epigenetic terms (“DNA methylation”, “chromatin”, “histone”, “enhancer”, “microRNA”, “non-coding RNA”, “epigenetic memory”). Additional searches used “biomarker”, “cfDNA”, “stool DNA”, “single-cell”, “spatial”, “DNMT inhibitor”, “HDAC”, “EZH2” and “BET”. Reference lists of recent reviews and key primary studies were hand-screened, and reviewer-identified 2025–2026 studies were assessed for relevance. The review prioritized peer-reviewed human IBD/CAC studies and direct mechanistic experiments; animal models and sporadic CRC or cell-line data were retained when they addressed a mechanistic gap, but are labeled as indirect evidence. Because this is a narrative rather than systematic review, no pooled meta-analysis or PRISMA-based effect estimate was performed.

### 2.2 Evidence hierarchy used throughout the review

Major mechanistic and translational claims are interpreted using five evidence contexts: (I) human CAC or IBD-associated dysplasia; (II) non-neoplastic human IBD mucosa, including field tissue; (III) inflammation-driven animal models such as AOM/DSS; (IV) sporadic CRC, organoid or cell-line evidence used for biological support; and (V) hypothesis-generating extrapolation. Evidence context is shown in **Tables 1A**, **B** and in the revised figures. A mechanistic observation is described as durable epigenetic memory only when the altered state persists after the initiating inflammatory stimulus has resolved or is removed; contemporaneous cytokine-dependent transcription is described as current inflammatory activity. The qualitative descriptors used in **Tables 1A**, **B** (for example, high, moderate or emerging) are narrative judgments of evidence directness and model concordance, not formal GRADE ratings.

**Table 1A:** Evidence-supported cytokine/epigenetic pathway nodes and model context.

Pathway node	Representative output	Model system	Evidence interpretation	Key reference(s)
IL-6/STAT3 → DNMT1 → SOCS3 promoter methylation	SOCS3 promoter hypermethylation; weakened negative feedback	Human UC-related dysplasia/cancer + experimental systems	High for this specific circuit; not universal	(17, 18)
NF-κB p65/DNMT3A → miR-148a repression	miR-148a promoter methylation; de-repression of upstream inflammatory nodes	Mouse colitis/tumorigenesis + mechanistic experiments	High in model; human CAC prevalence uncertain	(22)
STAT3 → miR-214 → PDLIM2/PTEN repression	Regulatory RNA-mediated reinforcement of NF-κB/AKT	Human UC association + mouse CAC experiments	Moderate-high; context dependent	(21)
Inflammatory oxidative damage → DNMT-containing complexes	Damage-associated recruitment of epigenetic machinery	Cellular/experimental cancer models	Indirect for CAC	(11)
Chronic inflammation → broad epigenetic program	Persistent methylation/chromatin alterations	Experimental intestinal inflammation/adenoma/CRC	Moderate; locus and model dependent	(12)
Resolved colitis → long-lived stem-cell chromatin memory	Persistent AP-1-associated accessibility; enhanced tumor growth after mutation	Mouse colitis with lineage/epigenomic tracking	Direct durable-memory evidence in mouse; human validation needed	(16)

**Table 1B:** Downstream phenotypes should be distinguished from pathway nodes.

Phenotype	Evidence context	Confidence/limitation	Key reference(s)
Proliferation/survival	Directly supported downstream of epithelial STAT3 and NF-κB in CAC models	High in animal CAC; variable human extrapolation	(7–9)
Stemness/plasticity	Supported by inflammatory/epigenetic programs; locus-specific human CAC evidence limited	Moderate	(12, 20)
Genomic instability	Inflammatory DNA damage plus epigenetically altered repair environment	Moderate; mixed direct/indirect evidence	(5, 11)
Immune evasion	STAT3/NF-κB and epigenetic regulation can shape suppressive microenvironments	Moderate; much evidence from broader CRC	(23, 24)
Therapy resistance	Epigenetic plasticity and cytokine survival signaling provide plausible resistance routes	Emerging in CAC; stronger broader CRC evidence	(20, 25)

## 3 Inflammation, genetic-epigenetic co-evolution and field cancerization

### 3.1 Recurrent injury and clonal selection

The chronically inflamed colon experiences spatially heterogeneous but recurrent oxidative stress, microbial products, prostaglandins, chemokines and cytokines. The immediate epithelial response is adaptive: damaged cells are replaced, crypt stem and progenitor cells proliferate, and barrier integrity is restored. Under persistent inflammatory pressure, however, cells that better tolerate ROS, replication stress or apoptosis gain a selective advantage. Epigenetic plasticity can alter cell state rapidly without a new DNA-sequence mutation, whereas mutations and copy-number changes can subsequently fix advantageous states (5, 11, 12).

Genetic and epigenetic evolution should therefore be evaluated together. Inflammation can increase mutational stress and alter the fitness landscape in which TP53, APC and other genetic events are selected; at the same time, methylation or chromatin changes can modify DNA-repair capacity, cytokine responsiveness and differentiation. The causal direction may differ between lesions, and cross-sectional tumor-versus-normal comparisons cannot determine which alteration occurred first. Longitudinal sampling is required to establish temporal order.

### 3.2 Operational definition of a field defect

We use ‘field cancerization’ operationally to mean a molecular abnormality detected in histologically non-neoplastic mucosa from an IBD colon that is spatially separated from an index dysplastic/cancer lesion and is enriched in patients with neoplasia compared with appropriately matched IBD controls. A convincing field marker should be defined by sampling location, inflammatory activity and cell composition, and ideally should precede or predict later neoplasia in longitudinal follow-up rather than merely track a current flare. Accelerated CpG methylation and inflammation-associated methylation have been described in non-neoplastic UC mucosa (13, 14). EYA4 methylation in non-neoplastic mucosa from UC patients with CRC provides a specific human example, although the cross-sectional design cannot by itself prove that the alteration preceded cancer (15).

### 3.3 Durable epigenetic memory versus transient inflammatory transcription

Cytokine signaling is often depicted as transient: ligand binds receptor, a kinase is activated, a transcription factor enters the nucleus and target genes are expressed. Chronic disease adds a temporal dimension. Repeated signaling can alter enhancer accessibility, promoter methylation and regulatory RNA programs, but persistence must be demonstrated after inflammatory activity subsides before the term ‘memory’ is applied. A recent mouse study provides unusually direct evidence: colonic stem cells retained inflammation-associated chromatin accessibility changes for more than 100 days after colitis resolution, and this memory enhanced tumor growth after oncogenic mutation through an AP-1-dependent program (16). This study establishes a principle of durable colitis memory in an experimental system, not proof that the same AP-1 program is present in every human CAC.

**Figure 1** therefore separates current cytokine signaling from durable epigenetic state and shows malignant phenotypes as a downstream layer influenced by inflammatory, signaling and epigenetic processes rather than by histone/chromatin regulation alone.

**Figure 1:**
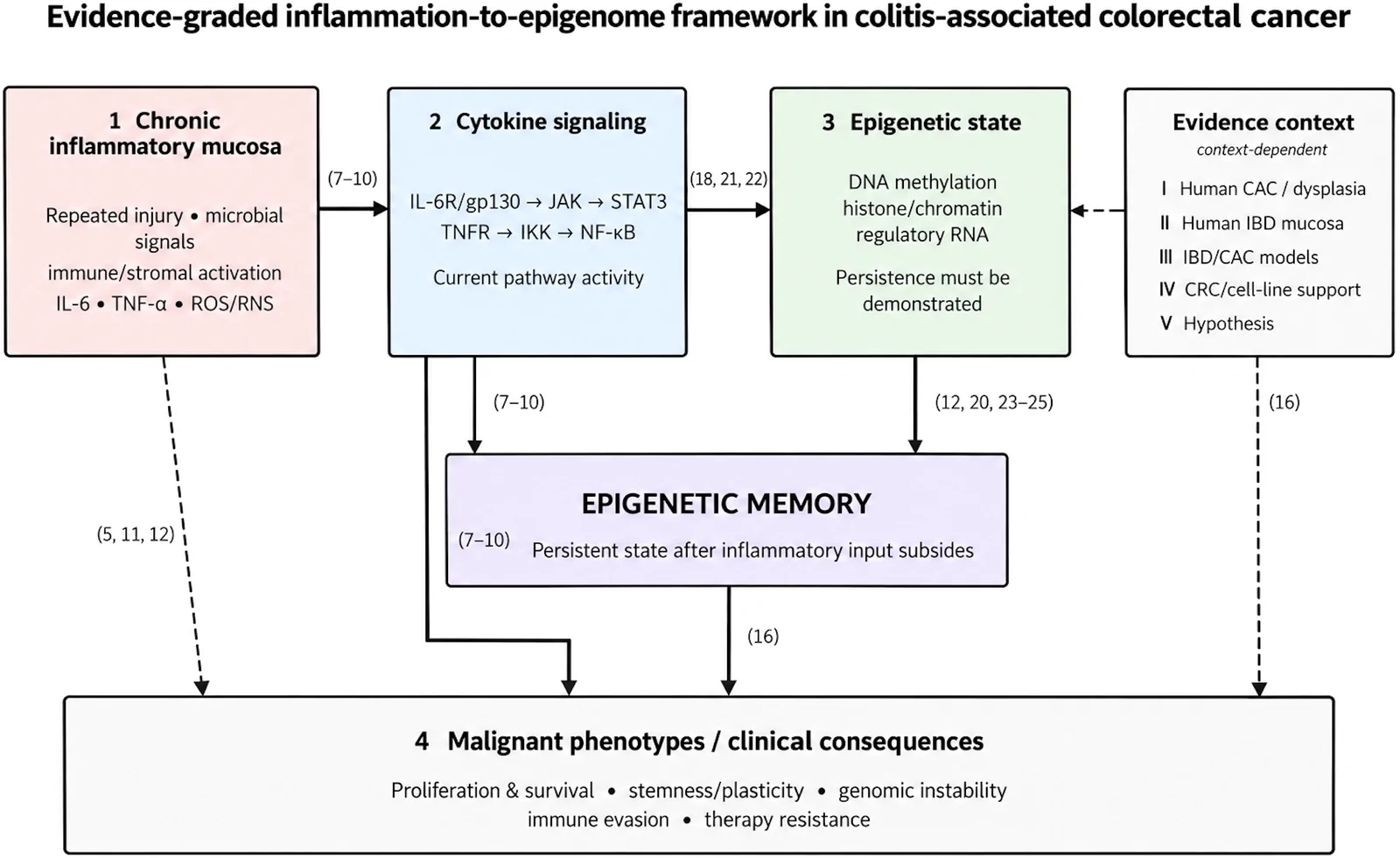
Evidence-graded inflammation-to-epigenome framework in CAC. The upper modules distinguish the chronically inflamed mucosa, receptor-proximal IL-6/JAK/STAT3 and TNF-α/NF-κB signaling, and epigenetic states. Malignant phenotypes are shown as a horizontal downstream layer because inflammatory exposure, signaling output and epigenetic remodeling can each contribute. Durable epigenetic memory is defined by persistence after inflammatory input subsides; direct evidence for long-lived post-colitis chromatin memory currently comes from experimental models (16). Representative supporting references are shown beside the corresponding arrows. Solid arrows denote direct/strong support in the stated model, whereas dashed arrows denote indirect or context-dependent support. Specific SOCS3, miR-148a and miR-214 circuits are reserved for **Figure 2**.

## 4 IL-6/JAK/STAT3 as a cytokine-to-chromatin pathway

### 4.1 Canonical and trans-signaling in the inflamed colon

IL-6 is produced by myeloid, stromal and activated lymphoid cells and, in some contexts, epithelial or tumor cells. Classical signaling requires membrane-bound IL-6 receptor, whereas trans-signaling uses soluble IL-6 receptor and expands the range of gp130-expressing responsive cells. Receptor engagement activates JAK kinases and promotes STAT3 phosphorylation, dimerization and nuclear accumulation. In mouse CAC models, persistent epithelial STAT3 supports survival and cell-cycle progression during repeated injury (7, 8). Human UC-associated neoplasia studies also show disease-related activation of the IL-6/STAT3/SOCS3 axis, but the magnitude and timing vary among lesions (17).

### 4.2 IL-6-DNMT1-SOCS3: a specific experimentally supported feedback circuit

Suppressor of cytokine signaling 3 (SOCS3) is a rapid negative-feedback regulator that binds JAK-family kinases, including JAK2, and limits phosphorylation of STAT3. In UC-related carcinogenesis, reduced SOCS3 expression can coincide with SOCS3 promoter hypermethylation and increased DNMT1 activity. Experimental IL-6 exposure increases DNMT1-dependent SOCS3 promoter methylation, and demethylating treatment can restore SOCS3 inducibility and reduce IL-6-driven proliferation (17, 18). These data provide a direct bridge between cytokine signaling and locus-specific methylation in UC-associated carcinogenesis.

The appropriate interpretation is not that SOCS3 promoter methylation is a canonical event in all CAC. Rather, it is a representative feedback mechanism demonstrated in a defined human disease context and experimental systems. Whether SOCS3 promoter methylation persists after inflammatory activity resolves, predicts future dysplasia in prospective cohorts, or is detectable with sufficient specificity in plasma or stool remains unproven.

### 4.3 STAT3 interactions with co-activators, repressors and enhancer state

STAT3 output depends on chromatin context and co-regulator recruitment. Outside CAC-specific systems, p300/CBP-mediated acetylation of STAT3 enhances DNA binding and transcriptional activity, providing a mechanistic basis for coupling cytokine signaling to acetylation-dependent regulation (19). Broader CRC and cancer studies also support interactions between STAT3 and Polycomb/HDAC-related programs, but direct cytokine-to-locus causality in human CAC remains incomplete (20). We therefore treat histone and enhancer mechanisms as biologically plausible and experimentally supported in related contexts, but at a lower CAC-specific evidence level than the SOCS3 circuit.

### 4.4 STAT3-controlled non-coding RNAs and downstream transcript networks

Cytokine-responsive microRNAs provide a route by which STAT3 can regulate a functional network of transcripts rather than a single target. In colitis-associated tumorigenesis, IL-6/STAT3 promotes miR-214 expression; miR-214 suppresses PDLIM2 and PTEN, thereby reinforcing NF-κB and PI3K/AKT signaling (21). This creates a cross-pathway feedback motif in which a STAT3-controlled non-coding RNA influences multiple downstream nodes. The miR-214 example, together with the miR-148a mechanism discussed below, shows that cytokine-epigenetic coupling extends beyond chromatin proteins.

## 5 TNF-α/NF-κB and cross-pathway feedback

### 5.1 TNFR signaling and sustained NF-κB activity

TNF-α is a central mediator of intestinal inflammation and a therapeutic target in IBD. TNFR engagement assembles receptor-proximal complexes containing RIPK1, TRAF proteins and cIAPs, leading under survival-permissive conditions to IKK activation, IκB degradation and nuclear NF-κB activity. If survival checkpoints fail or receptor complexes are remodeled, TNFR signaling can recruit death-pathway adaptors and induce apoptosis or necroptosis. In mouse CAC models, epithelial IKKβ deletion or TNF blockade reduces tumor formation, supporting a tumor-promoting role for sustained TNF/NF-κB signaling in those experimental settings (9, 10).

### 5.2 NF-κB-DNMT3A-miR-148a: transcriptional repression of a microRNA brake

miR-148a normally restrains several upstream components of inflammatory signaling. In inflammation-associated colon tumorigenesis, NF-κB p65 can cooperate with DNMT3A at the miR-148a promoter, increasing CpG methylation and reducing miR-148a expression; loss of miR-148a releases gp130, IKK-related components, IL1R1 and TNFR2 from repression (22). This mechanism is best described as transcriptional/epigenetic repression of the miR-148a gene that ultimately strengthens NF-κB and STAT3 pathway activity.

### 5.3 Cross-pathway wiring through regulatory RNAs and shared chromatin machinery

The miR-214 and miR-148a loops illustrate complementary modes of cross-talk. The miR-214 loop depends on transcriptional activation of a microRNA downstream of STAT3; the miR-148a loop depends on methylation-associated transcriptional repression of a microRNA downstream of NF-κB. Although the initiating events differ, both can increase pro-survival inflammatory signaling. Other regulatory RNAs may scaffold PRC2, LSD1, DNMTs or chromatin remodelers, but individual lncRNA mechanisms in human CAC remain less well validated and should be considered emerging rather than established drivers.

Repeated NF-κB occupancy is also associated with co-activator recruitment and enhancer acetylation in multiple inflammatory systems. In CAC, however, locus-specific maps linking TNF/NF-κB exposure to persistent human enhancer states remain incomplete. We therefore avoid describing such states as clinically measurable ‘enhancer addiction’ unless a specific assay and dependency have been validated.

### 5.4 Redox and metabolic coupling

Reactive oxygen and nitrogen species generated during chronic colitis produce DNA damage and can recruit complexes containing DNMT1, SIRT1 and Polycomb proteins to damaged chromatin, providing a mechanistic route from injury to later methylation changes (11). Metabolism can further influence methyltransferase, acetyltransferase and demethylase activity through S-adenosylmethionine, acetyl-CoA, alpha-ketoglutarate and redox state. Direct CAC-specific maps of these metabolic-epigenetic links remain limited, so they are presented as context-dependent mechanisms rather than universal CAC features (12).

**Figure 2** isolates three representative circuits and adds model-system labels so that experimentally supported examples are not visually presented with the same certainty as broader hypotheses.

**Figure 2:**
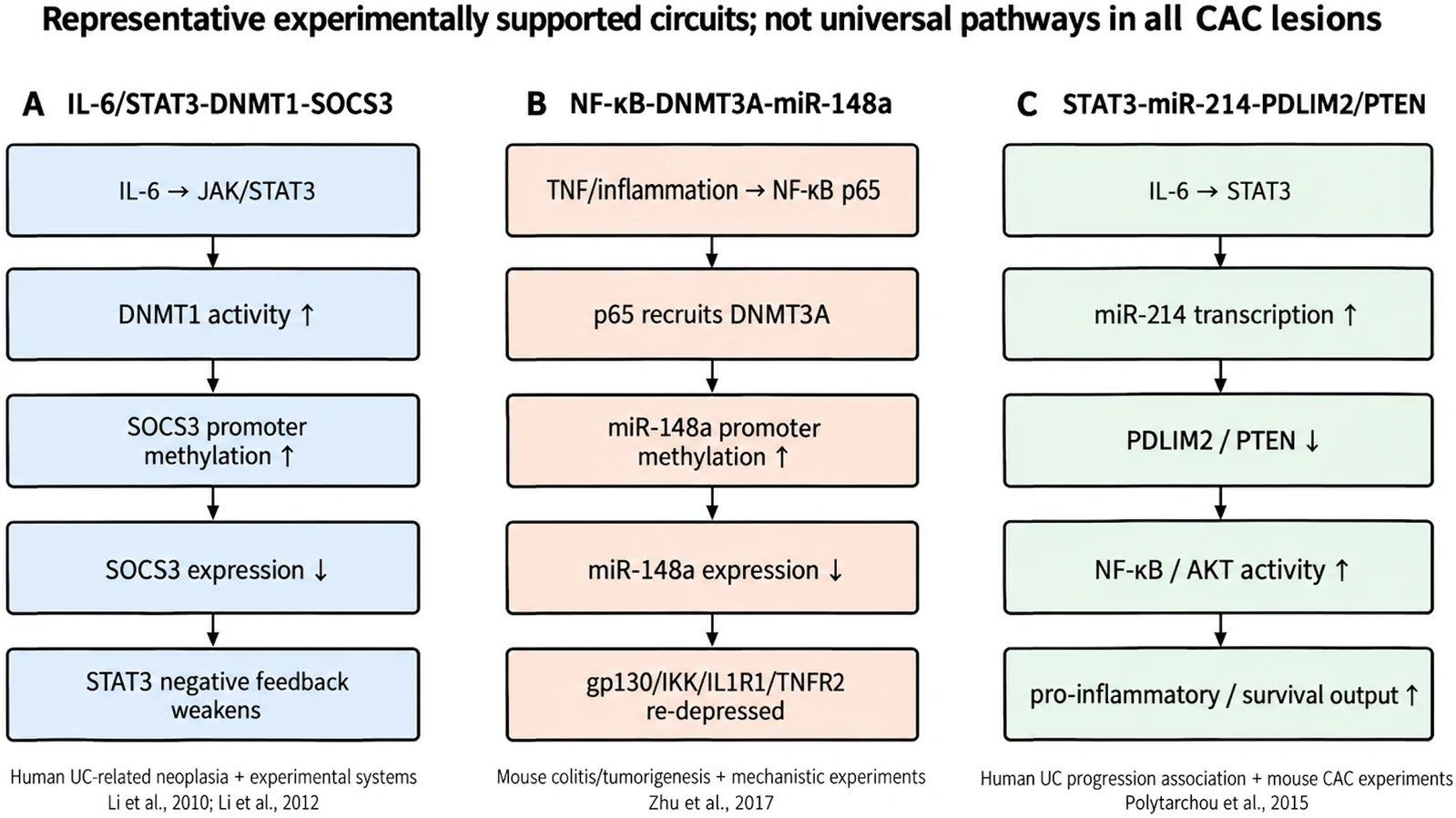
Representative experimentally supported circuits linking inflammatory signaling to epigenetic or regulatory persistence. **(A)** IL-6/STAT3-associated DNMT1 activity and SOCS3 promoter methylation in UC-related carcinogenesis (17, 18). **(B)** NF-κB p65/DNMT3A-associated miR-148a promoter methylation in colitis/tumorigenesis models (22). **(C)** STAT3-dependent miR-214 induction with PDLIM2/PTEN suppression in human UC progression studies and mouse CAC experiments (21). These circuits are examples, not universally active pathways in every patient or lesion.

## 6 Epigenetic outputs and functional consequences

### 6.1 DNA methylation and persistent field alterations

DNA methylation is chemically stable and measurable in tissue, blood and stool. In long-standing UC, methylation changes can accumulate in non-neoplastic mucosa (13, 14). Once promoter CpG islands of negative regulators are heavily methylated, silencing may persist through cell division. Persistence in a single cross-sectional specimen, however, is not equivalent to longitudinal proof that a marker preceded neoplasia. Candidate loci such as SOCS3 and EYA4 therefore require prospective validation before being described as risk predictors (15, 18).

### 6.2 Histone marks, enhancer remodeling and epigenetic memory

Histone modifications and chromatin accessibility respond rapidly to inflammatory signaling and may either resolve with inflammation or persist. Experimental chronic inflammation can establish an epigenetic program conserved in intestinal adenomas and CRC (12), while the 2026 colitis-memory study shows that a subset of accessibility changes can persist long after inflammation resolves in mice (16). These observations support a continuum from transient response to durable memory rather than a binary distinction between ‘inflammatory’ and ‘epigenetic’ states.

### 6.3 Epithelial plasticity, DNA repair, immune remodeling and treatment response

STAT3 and NF-κB support epithelial survival and regenerative programs in CAC models (7, 9). Epigenetic remodeling can alter differentiation thresholds and permit persistent plasticity, but terms such as ‘lineage infidelity’ are avoided here because they are difficult to operationalize independently of plasticity. In parallel, inflammatory DNA damage and epigenetic recruitment to damaged chromatin can couple genomic injury to altered repair states (11). These processes may interact with clonal selection rather than replace genetic evolution.

Chronic inflammation also reshapes immune and stromal compartments. Single-cell profiling of human UC demonstrates extensive epithelial-immune rewiring (24), and DNA-demethylating agents can induce viral-mimicry/interferon programs in CRC models (23). Such observations justify studying immune-epigenetic interactions, but they do not establish a CAC-specific therapeutic indication. Similarly, cytokine and chromatin programs may contribute to drug-tolerant states; direct CAC evidence remains less mature than evidence from sporadic CRC and other cancers (20).

## 7 What is established and what remains uncertain

The strongest mechanistic conclusion is that inflammatory signaling can alter epigenetic or regulatory machinery in defined colitis-associated systems. The SOCS3, miR-148a and miR-214 circuits provide specific examples; the 2026 colitis-memory study demonstrates that long-lived post-inflammatory chromatin states can exist in intestinal stem cells *in vivo* (16, 18, 21, 22). The weaker conclusion would be that these same circuits initiate or maintain every human CAC lesion; current evidence does not support that claim.

Three methodological gaps recur across the field. First, temporal causality is often unresolved because many studies compare established neoplasia with control tissue. Second, active inflammation can confound methylation, cfDNA and RNA measurements. Third, cell-of-origin is frequently uncertain in bulk tissue, blood and stool. These gaps define the requirements for translational work discussed below rather than serving as evidence that a candidate assay is already clinically actionable.

## 8 Translational outlook and future directions

### 8.1 Biomarker development: from biological plausibility to validated intended use

No epigenetic biomarker is currently validated for routine CAC surveillance. Candidate markers should first be assigned an intended use: (1) risk prediction of future dysplasia/CAC in patients without prevalent neoplasia; (2) early detection of prevalent dysplasia/CAC; or (3) longitudinal monitoring after treatment. These uses require different cohorts, time horizons and metrics. A marker that separates cancer from healthy controls does not establish future risk prediction, and a static tissue marker does not automatically provide a serial monitoring assay.

Human IBD studies nevertheless provide useful candidate evidence. TUBB6 methylation was identified in UC-associated neoplasia and validated in 71 biopsy samples; a *post-hoc* threshold yielded an AUC of 0.84, sensitivity 70.1% and specificity 98.6%, but independent prospective validation remains required (26). A methylated-miRNA panel was studied in 387 tissue specimens from 238 UC patients across two cohorts and showed strong discrimination within the study datasets, supporting a field-marker concept while also demonstrating age and sampling effects that must be controlled (27). Stool methylated BMP3 showed sensitivity of 60% for lesions <1 cm, 63% for low-grade dysplasia ≥1 cm and 81% for high-grade dysplasia/CRC at 91% specificity in an IBD study, illustrating both feasibility and the challenge of early-lesion sensitivity (28). EYA4 methylation in non-neoplastic mucosa is evidence for a field effect but not yet a validated longitudinal risk test (15). These candidate studies, together with the assay-performance and validation caveats discussed above, are summarized and compared in **Table 2**, which tabulates matrix, cohort, intended use, reference standard, reported performance, validation status and main false-positive/false-negative risks for each candidate CAC epigenetic biomarkers.

**Table 2:** Evidence and validation requirements for candidate CAC epigenetic biomarkers.

Candidate	Matrix	Cohort	Intended use	Reference standard	Reported performance	Validation status	Main false-positive/false-negative risks	Ref.
TUBB6 methylation	UC tissue/field biopsies	71 validation biopsies after discovery	Detection/field association	Histopathology-defined dysplasia/neoplasia	AUC 0.84; sens 70.1%; spec 98.6%; PPV 60.3%; NPV 99.1% (*post-hoc* threshold)	Secondary validation; no prospective external validation	Inflammation/sampling; prevalence-dependent PPV	(26)
Methylated miRNA panel (MIR1/9/124/137/34B/C)	Colorectal/rectal UC tissue	387 specimens, 238 UC patients, two cohorts	Risk enrichment/detection	UC dysplasia or CRC	Study-set AUC up to 0.97 for neoplasia; performance varies by specimen/location	Independent cohorts within study; prospective utility unproven	Age, segment, inflammation; field signal may reduce lesion specificity	(27)
mBMP3/mNDRG4/KRAS	Stool + lesion tissue	IBD lesions plus matched controls; independent stool cases/controls	Detection	Histologic IBD-associated neoplasia	mBMP3 stool sensitivity 60% (<1 cm), 63% LGD ≥1 cm, 81% HGD/CRC; specificity 91%	Single-center blinded phases; external prospective validation needed	Small/flat lesion sensitivity; shedding and pre-analytics	(28)
EYA4 methylation	Non-neoplastic UC mucosa	25 UC-CRC cases and 25 matched UC controls; DNA evaluable in 20/20	Field association/candidate risk marker	Concurrent UC-CRC status	Field enrichment demonstrated; longitudinal prediction not established	Cross-sectional matched study	Cannot distinguish antecedent field from cancer-associated field	(15)
SEPT9/average-risk stool DNA	Plasma or stool	Large average-risk CRC cohorts	Technical precedent only	Average-risk CRC screening	Validated for sporadic CRC screening, not CAC	Not a CAC validation stage	Active inflammation and different prevalence/spectrum can alter performance	(29–31)

Average-risk CRC assays such as plasma SEPT9 or multitarget stool DNA demonstrate technical feasibility for methylated-DNA detection but should not be extrapolated to CAC surveillance (29–31). Active IBD without dysplasia, quiescent IBD, low-grade dysplasia, high-grade dysplasia, CAC and sporadic CRC should be analyzed as distinct comparator groups. Clinically meaningful endpoints should include advanced neoplasia, invisible dysplasia, interval cancer, colonoscopy yield or a prespecified change in surveillance interval rather than an undefined ‘molecularly advanced field’ (4, 32).

Confounding variables must be prespecified: disease activity and extent, age, medications, primary sclerosing cholangitis, prior dysplasia and bowel preparation can influence tissue, circulating and stool-derived signals. Liquid-biopsy studies should report plasma versus serum, collection tube, processing time, storage conditions, cfDNA input, bisulfite versus enzymatic or sequencing method, analytical limit of detection and batch-control procedures. Discovery studies should preferentially collect matched tissue, blood and stool to permit tissue-of-origin analysis and deconvolution of epithelial, immune and stromal contributions.

Future multimodal models should integrate complementary clinical and molecular domains transparently rather than relying on opaque feature accumulation. Methodological examples from gastric cancer—not direct evidence for CAC—illustrate several relevant principles: integration of complementary data types (33), interpretable multimodal fusion with external/prospective validation (34), broad independent benchmarking and stability assessment (35, 36), and separation of static tissue risk assessment from serial liquid-biopsy monitoring (37, 38). A recent prospective multimodal radiopathomics study likewise illustrates the value of predefined external and prospective validation, although its disease setting and endpoint differ from CAC surveillance (39). These studies are cited for validation design principles only.

### 8.2 Therapeutic opportunities: evidence level and safety boundaries

No epigenetic drug is currently established for CAC prevention. Therapeutic discussion should therefore distinguish established IBD anti-inflammatory practice from preclinical epigenetic hypotheses and from treatment of established cancer. Effective inflammatory control is clinically important in IBD, but anti-TNF and JAK inhibitors should not be described as proven epigenetic chemoprevention. Observational anti-TNF data are vulnerable to confounding by indication and cumulative inflammatory burden, and there is no direct evidence that these agents erase a pre-existing high-risk epigenetic field (3, 40).

DNMT inhibition reduces inflammation-induced colon tumorigenesis in preclinical models and provides proof of principle that methylation can be functionally relevant (41, 42). HDAC, EZH2 and BET-directed approaches have mechanistic support in CRC or other cancers, but CAC-specific human evidence is limited (20). JAK inhibitors such as tofacitinib and upadacitinib are established treatments for UC, not established CAC-prevention drugs (43, 44). Direct STAT3 or NF-κB inhibition remains investigational for CAC. **Table 3** classifies the principal therapeutic concepts along the inflammation–epigenetics axis (optimized IBD inflammatory control, JAK inhibition, DNMT inhibition, HDAC inhibition, EZH2 inhibition, BET inhibition, and epigenetic therapy plus immune checkpoint blockade) by evidence level, human CAC evidence, route/duration context, toxicity and IBD-specific safety, and interpretive status.

**Table 3:** Therapeutic concepts along the inflammation-epigenetics axis, classified by evidence level.

Strategy	Evidence level	Human CAC evidence	Route/duration context	Toxicity/IBD-specific safety	Interpretation	Ref.
Optimize IBD inflammatory control	Established IBD practice; indirect prevention rationale	Human IBD; no proof of epigenetic-field erasure	Biologics/oral agents; chronic maintenance varies by drug	Infection and drug-specific adverse effects; confounding by indication in prevention analyses	Not an epigenetic chemoprevention claim	(3, 40)
JAK inhibition	Established for moderate-severe UC	Human IBD RCTs; no dedicated CAC prevention efficacy	Oral, generally daily continuous therapy per indication	Infection, thrombosis/MACE and other label-specific risks; immune effects	Not established for CAC prevention or treatment	(43, 44)
DNMT inhibition	Preclinical CAC proof of principle	Inflammation-driven animal models; CRC cell studies	Systemic agents in oncology; prevention duration undefined	Myelosuppression, off-target demethylation, lack of locus specificity	No established CAC prevention use	(23, 41, 42)
HDAC inhibition	Mechanistic/broader oncology evidence	Mostly non-CAC; some approved hematologic indications	Oral or IV depending agent; oncology schedules	Broad transcriptional effects, cytopenias, fatigue, GI/cardiac effects vary	CAC clinical evidence insufficient	(20)
EZH2 inhibition	Mechanistic/selected oncology indications	No established CAC-specific benefit	Oral for approved non-CAC indications	Drug-specific hematologic and secondary malignancy warnings	Biomarker-defined CAC trials would be required	(20)
BET inhibition	Investigational	Preclinical/early oncology studies; no established CAC use	Usually oral investigational regimens	Thrombocytopenia and therapeutic-index limitations	Hypothesis only in CAC	(20)
Epigenetic therapy + ICI	Early clinical evidence in MSS CRC, not CAC-specific	Advanced cancer context	Trial-specific	IBD flare/immune-mediated colitis; approximately 40% relapse estimate in pre-existing IBD	Not a prevention strategy	(25, 45)

For established cancer, epigenetic therapy plus immune checkpoint blockade remains experimental in most MSS CRC settings (25). Pre-existing IBD creates a specific safety concern: a meta-analysis of 193 patients with IBD treated with checkpoint inhibitors estimated an IBD relapse rate of approximately 40% (95% CI 26%-55%); among those relapsing, about 76% required corticosteroids and 37% required biologic therapy (45). Treatment of an established malignancy must therefore be distinguished from prevention in non-dysplastic colitis, where such risk would be unacceptable without compelling evidence.

### 8.3 Single-cell, spatial and perturbation technologies: a translational roadmap

Single-cell and spatial technologies can help resolve cell-of-origin, but a clinically useful roadmap must treat the patient—not the number of profiled cells—as the unit of validation. Discovery cohorts should collect paired samples from active IBD without dysplasia, quiescent IBD, dysplasia and CAC, with multiple spatially defined biopsies per patient and matched blood/stool when biomarker translation is intended. Patient-level sample-size calculations should be prespecified from expected effect sizes; pilot datasets with tens of patients per state may generate hypotheses, whereas clinical validation will generally require independent multicenter cohorts in the hundreds rather than merely deeper sequencing of a few specimens.

Specimen handling should be matched to the intended assay. Fresh or rapidly cryopreserved tissue is preferred for scRNA-seq and many single-cell ATAC workflows; nuclei-based methods can extend analysis to frozen tissue, while FFPE-compatible spatial transcriptomic and targeted protein methods may provide greater compatibility with routine pathology but different resolution and molecular breadth. Spatial resolution should be selected to distinguish epithelial crypts, stromal niches and immune aggregates rather than maximized without a clinical question. Tissue preservation, ischemia time, batch, sequencing depth, platform version and segmentation/deconvolution algorithms should be recorded prospectively (46).

A practical analysis plan would use single-cell data to define cell states, spatial data to anchor those states to dysplasia boundaries and inflammatory niches, and perturbation models to test causality. Patient-derived organoids can assess whether candidate methylation or accessibility states persist after removal from the inflammatory environment; co-culture and CRISPR perturbation can then test reversibility. Translation should be judged by a predefined decision: for example, whether a spatial signature increases yield of targeted biopsies, whether a cell-type-resolved marker improves discrimination of active IBD from dysplasia, or whether a perturbation predicts response to a therapy. Descriptive atlases without a decision endpoint should not be presented as clinical workflows (24, 46).

## 9 Conclusions

CAC is best viewed as the product of chronic inflammatory exposure interacting with genetic, clonal and epigenetic evolution. IL-6/JAK/STAT3 and TNF-α/NF-κB provide experimentally tractable examples of cytokine-to-epigenome coupling, but current evidence does not establish them as universal initiating drivers of human CAC. The IL-6-DNMT1-SOCS3 promoter circuit, NF-κB-DNMT3A-miR-148a repression and STAT3-miR-214-PDLIM2/PTEN signaling are specific supported examples whose prevalence and persistence must be tested across human lesions (18, 21, 22). Long-lived post-colitis chromatin memory is now directly demonstrated in an experimental stem-cell system, strengthening the biological basis for studying persistence while underscoring the need for human longitudinal validation (16).

The translational priority is therefore not to declare a single biomarker or epigenetic therapy ready for CAC management. It is to design studies that distinguish risk prediction from early detection and monitoring, use clinically relevant comparator groups, standardize pre-analytics, resolve cell-of-origin, and validate models externally and prospectively. The same discipline should be applied to therapeutic concepts: inflammatory control is established IBD care, whereas epigenetic intervention for CAC prevention remains preclinical or hypothesis-generating. Evidence-graded integration of clinical, molecular and longitudinal data offers a realistic route from mechanism to disease management without conflating biological plausibility with clinical validation.
