# Supplementary material for: Crosstalk between IL-6/JAK/STAT3 and TNF-α/NF-κB signaling in colitis-associated colorectal cancer: epigenetic reprogramming, evidence boundaries, and opportunities for disease management
Source: Front Immunol. 2026 Oct 26;17:1979583. doi: 10.3389/fimmu.2026.1979583 (PMC13746221; doi:10.3389/fimmu.2026.1979583)
Supplement: Supplementary file 1 [file Table4.docx]

# Editable Tables for Submission

Table 1. Major cytokine-linked epigenetic mechanisms in colitis-associated colorectal cancer.

| Inflammatory input | Epigenetic effector | Representative epigenetic output | Biological consequence | Current evidence |
| --- | --- | --- | --- | --- |
| IL-6/JAK/STAT3 | DNMT1 induction/activity; STAT3 acetylation; p300/CBP recruitment | SOCS3 promoter hypermethylation; active inflammatory/survival enhancers | Persistent STAT3 signaling, survival, proliferation, field susceptibility | Direct mechanistic evidence in UC-related carcinogenesis; broader support in CRC models |
| TNF-α/NF-κB | p65 interaction with DNMT3A; p300/CBP; HDAC and chromatin-remodeling complexes | miR-148a promoter methylation; inflammatory enhancer accessibility | Amplified TNFR/IKK/gp130 signaling, inflammatory memory | Strong mechanistic evidence for miR-148a circuit in colitis/tumorigenesis; locus-specific histone effects are context dependent |
| STAT3-NF-κB crosstalk | Shared co-activators; reciprocal cytokine induction; enhancer co-occupancy | Stable high-accessibility inflammatory regulatory elements | Pathway redundancy, plasticity, immune suppression | Biologically well supported; CAC-specific enhancer maps remain incomplete |
| ROS/repair response | DNMT1 recruitment with DNA-damage/repair machinery | Inflammation-associated CpG hypermethylation | Tumor-suppressor silencing and clonal fixation | Preclinical inflammation-driven colon tumor models |
| Polycomb/HDAC axis | EZH2/PRC2, HDACs, BET readers | H3K27me3 gain at repressed loci; altered acetylation at active enhancers | Stemness, lineage infidelity, drug resistance | Strong CRC evidence; direct cytokine-to-locus causality in CAC requires deeper profiling |
| TET/5hmC axis | Inflammatory and metabolic regulation of TET activity | Loss or redistribution of 5hmC | Reduced epigenetic plasticity toward normal differentiation | Emerging; likely important but under-characterized in human CAC |

Abbreviations: DNMT, DNA methyltransferase; HDAC, histone deacetylase; PRC2, Polycomb repressive complex 2; TET, ten-eleven translocation dioxygenase; 5hmC, 5-hydroxymethylcytosine.

Table 1 emphasizes that the epigenetic machinery downstream of inflammatory cytokines is highly convergent. DNMTs, acetylation-dependent co-activators, HDACs, Polycomb complexes and chromatin-remodeling proteins are not exclusive to either STAT3 or NF-κB; instead, their recruitment depends on genomic context, cell state and the duration of inflammatory signaling. The practical implication is that the most informative mechanistic biomarkers are likely to combine pathway activity with a locus-specific methylation or chromatin readout rather than rely on expression of a single epigenetic enzyme. The evidence column also highlights an important distinction for interpretation: some nodes, such as the IL-6-DNMT1-SOCS3 and NF-κB-DNMT3A-miR-148a circuits, have direct mechanistic support in colitis-associated models, whereas broader histone and TET effects remain more context dependent and require deeper CAC-specific validation.

Table 2. Candidate epigenetic biomarker strategies for CAC surveillance.

| Biomarker strategy | Sample type | Biological rationale | Potential use | Key limitation |
| --- | --- | --- | --- | --- |
| SOCS3 promoter methylation | Tissue; potentially cfDNA/stool after assay development | Directly linked to IL-6/DNMT1 and loss of STAT3 negative feedback | Risk-field and pathway-activity marker | Requires prospective longitudinal validation and assay optimization outside tissue |
| miR-148a methylation/expression | Tissue, plasma/exosomes, stool | Mechanistically links NF-κB/DNMT3A to amplification of STAT3/NF-κB | Mechanistic risk signature | Need cell-of-origin resolution and threshold definition |
| miR-214 / inflammatory miRNA panels | Plasma, serum, stool, tissue | Reflects IL-6/STAT3-NF-κB feedback and inflammatory progression | Dynamic monitoring | Inflammation can confound cancer specificity |
| SEPT9/SDC2 and CRC methylation panels | Plasma and/or stool | Validated principle for CRC detection | Adjunct early detection | Not CAC specific; early lesion sensitivity and IBD specificity need dedicated study |
| CAC-specific methylome signatures | Plasma cfDNA, stool, tissue | Capture field defects and lesion-associated methylation | Risk stratification / early dysplasia detection | Requires large discovery + independent prospective validation cohorts |
| Chromatin accessibility / histone signatures | Biopsy; emerging cf-nucleosome approaches | Reflects active regulatory state and cytokine-responsive enhancers | Mechanistic classification / therapy selection | Current clinical assays are immature; tissue heterogeneity is substantial |
| Multimodal cfDNA + miRNA + inflammation score | Blood ± stool | Integrates epigenetic state with disease activity and tissue origin | Longitudinal precision surveillance | Needs standardized pre-analytics, serial cohorts and clinically defined action thresholds |

Table 2 shows why no single specimen type is likely to solve CAC surveillance. Plasma permits repeated minimally invasive sampling but can dilute early mucosal signals; stool is anatomically well positioned to capture exfoliated colorectal material but is affected by pre-analytical variation and microbial background; tissue provides spatial specificity but is invasive and subject to sampling error. Accordingly, the most credible development path is a multimodal, longitudinal strategy that combines stable epithelial methylation markers with inflammatory or extracellular-vesicle RNA features and established clinical risk variables. Such panels must be prospectively tested against the clinically difficult distinction between active non-neoplastic IBD and molecularly advanced dysplasia or CAC, rather than only against healthy controls.

Table 3. Therapeutic opportunities along the inflammation-epigenetics axis.

| Strategy | Mechanistic rationale | Best-fit disease stage | Opportunity | Principal caution |
| --- | --- | --- | --- | --- |
| Optimize IBD inflammatory control | Reduces cumulative IL-6/TNF/ROS exposure | Long-standing colitis before dysplasia | Biologically central and clinically available | Does not reliably erase established epigenetic memory; drug-specific chemoprevention evidence varies |
| JAK inhibition / STAT3-directed therapy | Suppresses ongoing IL-6-family signaling and STAT3 transcription | Active cytokine-dependent field or established tumor | JAK inhibitors available in IBD; direct STAT3 inhibitors mostly investigational oncology agents | Immune effects, pathway redundancy, need to identify STAT3-dependent tumors |
| TNF/NF-κB pathway modulation | Reduces inflammatory input and NF-κB enhancer activation | Inflammatory phase; selected tumor contexts | Anti-TNF established for IBD; direct NF-κB blockade not established for CAC | TNF/NF-κB have context-dependent protective functions; no proven CAC chemoprevention by anti-TNF alone |
| DNMT inhibition | Reverses aberrant methylation; restores tumor suppressors; viral mimicry | High-risk epigenetic field concept / resistant tumor | Strong preclinical proof of principle | Toxicity and lack of locus specificity limit prevention use |
| HDAC inhibition | Restores acetylation and pro-apoptotic transcription | Epigenetically repressed, therapy-resistant tumor | Several approved agents in hematologic malignancy; solid-tumor combinations under study | Broad transcriptional effects and dose-limiting toxicity |
| EZH2/PRC2 inhibition | Reverses H3K27me3-mediated repression | EZH2-high stem-like or immune-cold states | Mechanistically attractive, biomarker-dependent | CAC-specific validation limited |
| BET inhibition | Disrupts acetylated inflammatory enhancers/super-enhancers | Enhancer-addicted tumor states | Strong mechanistic rationale | Therapeutic index and adaptive resistance |
| Epigenetic drug + immunotherapy | Restores antigen presentation/IFN programs and counters immune escape | Advanced CAC with suppressive microenvironment | High translational interest | IBD flare risk; requires multidisciplinary monitoring and careful patient selection |

Table 3 underscores the stage dependency of therapeutic translation. Anti-inflammatory treatment is most rational before a self-sustaining epigenetic program is fully established, whereas chromatin-directed therapy is more likely to be useful when a measurable epigenetic dependency contributes to persistence, plasticity or drug resistance. Because DNMT and HDAC inhibitors act broadly, their use in IBD-associated cancer should be guided by pharmacodynamic biomarkers and balanced against effects on mucosal repair and immune function. The table therefore supports a mechanism-selected rather than indiscriminate combination strategy: the inflammatory driver, the epigenetic lesion and the anticipated escape pathway should ideally be defined in the same tumor or high-risk field before combined pathway and epigenetic intervention is attempted.
